# Deciphering the Transcriptomic Heterogeneity of Duodenal Coeliac Disease Biopsies

**DOI:** 10.3390/ijms22052551

**Published:** 2021-03-04

**Authors:** Johannes Wolf, Edith Willscher, Henry Loeffler-Wirth, Maria Schmidt, Gunter Flemming, Marlen Zurek, Holm H. Uhlig, Norman Händel, Hans Binder

**Affiliations:** 1 Department of Laboratory Medicine at Hospital “St. Georg” Leipzig, 04129 Leipzig, Germany; Johannes.Wolf@sanktgeorg.de; 2 Immuno Deficiency Centre Leipzig (IDCL) at Hospital St. Georg Leipzig, Jeffrey Modell Diagnostic and Research Centre for Primary Immunodeficiency Diseases, 04129 Leipzig, Germany; 3 IZBI, Interdisciplinary Centre for Bioinformatics, University Leipzig, Härtelstr. 16–18, 04107 Leipzig, Germany; edith.willscher@uk-halle.de (E.W.); wirth@rz.uni-leipzig.de (H.L.-W.); schmidt@izbi.uni-leipzig.de (M.S.); 4 Paediatric Gastroenterology Unit, University Hospital for Children and Adolescents, 04103 Leipzig, Germany; gunter.flemming@medizin.uni-leipzig.de; 5 Children’s Hospital of the Clinical Centre “Sankt Georg”, 04129 Leipzig, Germany; marlen.zurek@sanktgeorg.de (M.Z.); norman.haendel@sanktgeorg.de (N.H.); 6 Translational Gastroenterology Unit, Oxford NIHR Biomedical Research Centre, Experimental Medicine, Department of Paediatrics, University of Oxford, John Radcliffe Hospital, Oxford OX4 2PG, UK; holm.uhlig@ndm.ox.ac.uk

**Keywords:** molecular subgroups, gene expression signatures, villous atrophy, immune cell de-convolution, machine learning, self-organizing maps, personalized diagnostics

## Abstract

Coeliac disease (CD) is a clinically heterogeneous autoimmune disease with variable presentation and progression triggered by gluten intake. Molecular or genetic factors contribute to disease heterogeneity, but the reasons for different outcomes are poorly understood. Transcriptome studies of tissue biopsies from CD patients are scarce. Here, we present a high-resolution analysis of the transcriptomes extracted from duodenal biopsies of 24 children and adolescents with active CD and 21 individuals without CD but with intestinal afflictions as controls. The transcriptomes of CD patients divide into three groups—a mixed group presenting the control cases, and CD-low and CD-high groups referring to lower and higher levels of CD severity. Persistence of symptoms was weakly associated with subgroup, but the highest marsh stages were present in subgroup CD-high, together with the highest cell cycle rates as an indicator of virtually complete villous atrophy. Considerable variation in inflammation-level between subgroups was further deciphered into immune cell types using cell type de-convolution. Self-organizing maps portrayal was applied to provide high-resolution landscapes of the CD-transcriptome. We find asymmetric patterns of miRNA and long non-coding RNA and discuss the effect of epigenetic regulation. Expression of genes involved in interferon gamma signaling represent suitable markers to distinguish CD from non-CD cases. Multiple pathways overlay in CD biopsies in different ways, giving rise to heterogeneous transcriptional patterns, which potentially provide information about etiology and the course of the disease.

## 1. Introduction

Coeliac Disease (CD) is a systemic autoimmune-mediated enteropathy triggered by gluten peptides from wheat and closely related cereals [1,2]. The ingestion of gliadin in genetically susceptible individuals may lead to intraepithelial lymphocytosis, crypt hyperplasia, villous atrophy and a chronic inflammatory cell infiltrate in the lamina propria [3]. CD is one of the most prevalent food intolerance with a prevalence of currently 1.4%, and a historic increase over the last three decades [4,5]. CD patients carry at least one allele of HLA-DQ2 or HLA-DQ8, which define a specific type of membrane receptors on antigen presenting cells (APCs), responsible for the body’s first line of defense. After binding gliadin, APCs may activate an inflammatory response [6]. However, CD-associated HLA-DQ molecules are not the only trigger for disease development since similar HLA-haplotypes occur in approximately 40 percent of the general population [7]. The diagnosis of CD is challenging and relies on the assessment of a highly variable clinical status, antibody measurements, histological evaluation of intestinal biopsies. The latter is classified by Marsh staging, which is based on assessment of villous atrophy and crypt hyperplasia as well as the count of intraepithelial lymphocytes (IELs). Furthermore, the response to gluten-free diet, which is nowadays the only treatment for CD, is an additional clinical criterion. For children and adolescents with suspected CD, biopsies are avoidable for establishing a CD diagnosis if IgA antibodies against tissue transglutaminase reach more than a tenfold excess compared with the upper limit of normal level (≥10 × ULN) and if IgA Endomysium antibodies show positivity in a second blood sample [8]. Otherwise, this diagnostic approach isn’t recommended for adults. Upper endoscopy with small bowel biopsy serves to establish the diagnosis for most adult patients and also for children with only mild elevated antibodies (<10 × ULN) with suspected coeliac disease. Previously regarded as a gold standard of diagnostics, unsatisfactory inter-observer agreement was reported between histopathologists [8,9,10,11]. The use of validated standard operating procedures (SOPs) with correct orientation and cutting of the duodenal specimen is critical for an accurate interpretation of the mucosal architectural [11,12]. At this point transcriptome studies of duodenal tissue may shed light on the diagnosing challenge of coeliac disease.

Fundamental transcriptomic research is focusing on small-intestinal samples of CD patients and on the cell types present therein in order to uncover pathogenic pathways that are altered in the small intestine of CD patients. These techniques are expected to open new options for CD diagnostics and monitoring. Particularly, transcriptome studies of intestinal biopsies of CD patients have revealed genes and pathways that are altered in the disease and might serve as biomarkers for small intestinal damage and function. Previous gene expression studies were performed on the purpose of Marsh-score discrimination [13], to characterize villous atrophy [14,15,16], epithelial destruction [17], upregulation of immunity [18,19], differences of gene expression of CD in adults and children [20] and of genes mutated in CD [19,21,22] (see also [23] for a review).

Overall, these studies indicate correspondence between established histological and serum markers on one hand and molecular transcriptomic signatures on the other hand. However, heterogeneity of transcriptomic patterns is extensive and requires further clarification. A systematic view on the relations between transcriptomic indications of villi atrophy, crypt dysplasia, signs of inflammation, immune cell infiltration and other factors in order to make it usable for an improved management for CD patients is still lacking.

We have performed a detailed analysis of the transcriptional heterogeneity of duodenal biopsies from 24 Patients with CD, 20 patients without CD, and one patient with unclear diagnosis recruited as part of the ABCD (Antibody diagnostics in pediatric Coeliac Disease) study in two children hospitals in Leipzig (Saxonia, Germany). We applied a comprehensive analysis and visualization strategy based on self-organizing maps (SOM) machine learning and downstream functional analysis [24,25,26].

In the first part of the paper the cases were stratified by means of unsupervised clustering and characterized based on previous knowledge to obtain a comprehensive view on CD as seen by transcriptomics. In the second part, functional analysis is further refined by extracting information about immune cell infiltration and by high-resolution personalized portrayal of the transcriptomic landscape of CD. In the third part, we address the issue of feature selection for diagnosing CD in our data and we present a novel tool for exploring the data under the specific perspective of interested scientists called SOM-browsing.

## 2. Results

### 2.1. Clustering Identifies Three Transcriptional Subgroups of CD Patients

**Figure 1:**
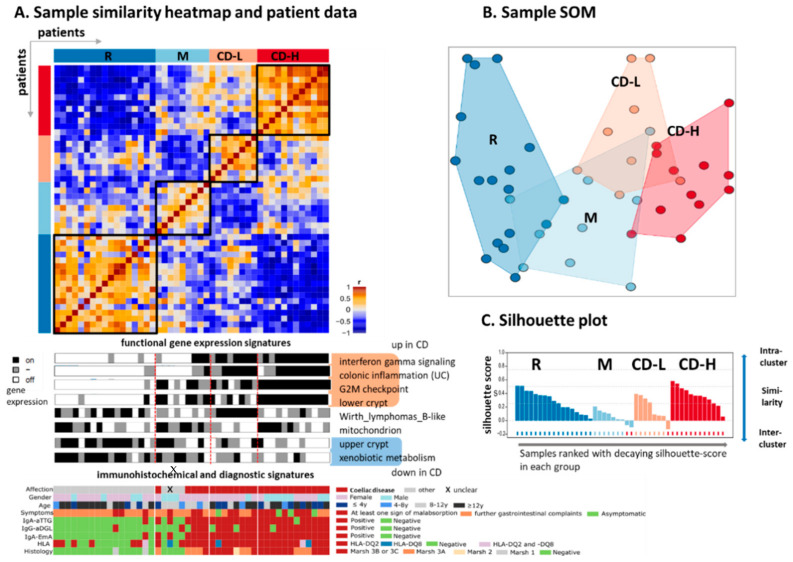
Typing of the patients. (**A**) The pairwise correlation heatmap visualizes cross-correlations between the biopsies, which split into four expression groups: disease controls serving as reference (R), mixed (M), less severe ‘coeliac disease low’ (CD-L) and severe ‘CD-high’ (CD-H). Gene expression levels of characteristic functional signatures are shown as black-white ‘barcodes’ below the heatmap (see Figure A2, Figure A3 and Figure A4 for details). They upregulate (black color) specifically in some of the biopsy groups. The samples within each group were ranked with increasing expression of the gene signature “colonic inflammation” [27]. High expression levels of this signature are associated with diagnosed CD together with positive (auto)-antibodies and histology. X = patient with unclear diagnosis. (**B**) The sample diversity map (sample SOM, see also Figure A1) visualizes similarity relations between the transcriptomes of the different biopsies (circles) in two dimensions. The distances between the samples scale with their mutual similarity, which was estimated in terms of correlation coefficients between their expression portraits (see below). (**C**) The silhouette plot further supports clustering: Positive silhouette scores indicate stable clustering of the respective samples within the cluster environment chosen (see Materials and methods). Negative scores indicate closer similarity to another cluster.

We analyzed gene expression data of duodenal probes from 24 children and adolescents aged between 5 months and 18 years with active CD (Table 1). Another 21 patients of the same age range without CD diagnosed but with diverse intestinal afflictions served as controls. One further patient could not be clearly diagnosed (Table 1). Gene expression data of each patient were transformed into self-organizing maps (SOM) transcriptomic portraits providing “personalized” images of their gene expression states (see below). Clustering of SOM-transformed metagene data provides four major groups of samples: the first group contains only disease control samples referred as reference group (R). A second M-group (mixed, 31% of all samples) collects a mixture of four control, four CD samples and one with unclear diagnosis while the remaining two groups are exclusively formed by CD-samples. They were assigned as CD-low (CD-L, n = 8, 27%) and CD-high (CD-H, n = 12, 41%). Based on similarity analysis by means of the pairwise correlation heatmap (Figure 1A) and sample-SOM (Figure 1B), one sees that CD-L and M group transcriptomes show intermediate features combining that of the reference and of the CD-H groups. Biopsies in each of the groups were ranked with increasing expression levels of the gene signature ‘colonic inflammation’ extracted from ulcerative colitis biopsies for better representation [27,28]. Other key functional expression signatures show either activation or deactivation in the different groups (see ‘barcodes’ in Figure 1A and next subsection for details).

The cases in groups CD-H and CD–L show high levels of IgG-antibodies against deamidated gliadin peptides (IgG-aDGL, ≥25 U/mL) and of IgA class antibodies against tissue transglutaminase (IgA-TTG, ≥20 U/mL). They also show high levels of IgA endomysial antibodies (IgA-EmA, ≥1:10 U/mL, all *p* < 0.01 in Fisher’s exact test) and enrich the HLA-DQ2 type. CD-H patients have the highest marsh stages (3B and 3C) (*p* < 0.01, Fisher’s exact test) and highest median age (12.5 years) among all three groups with CD patients. A percentage of 25% of the patients of CD-H suffer from diabetes type 1. Seven out of eight CD-L patients are females and most of them (87.5%) show signs of malabsorption. Malabsorption is also typical for all patients of group M and except one, all other (88.9%) had gastrointestinal afflictions but none of them had diabetes. Clinical and histological characteristics of subgroups are summarized in Table 1. Taken together, according to transcriptomic characteristics we identified one reference group and three groups of CD-patients differing from patients with other gastrointestinal afflictions according to transcriptome characteristics of the duodenum. The groups CD-H and CD-L suggest different levels of CD severity both showing symptoms of inflammation by exhibiting high titers of IgA-aTTG and IgA-EmA and high Marsh stages compared to groups M and R (*p* < 0.01, Fisher’s exact test). Alternatively we have in view the option that CD-L mimicries lower severity because slightly varying tissue composition of the biopsies.

### 2.2. Expression Signatures Characterize the Functional State of the Small Intestine Epithelium

**Figure 2:**
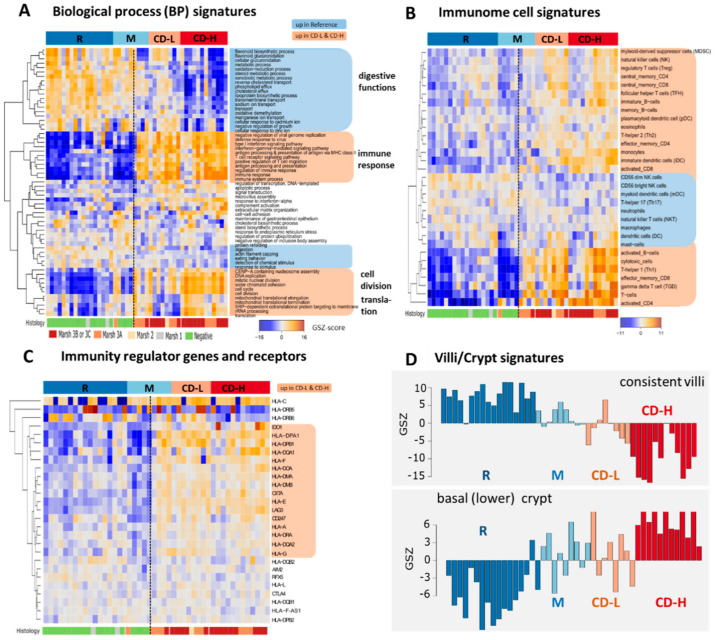
Gene set expression signatures characterize the functional context of CD-types: Expression heatmaps of, (**A**) gene sets referring to GO category biological process (BP) and, (**B**) of ‘Immunome’ related gene sets taken from [30] reveal functional categories up- or down-regulated in CD-H and CD-L (marked in red or blue, respectively). The former ones include different gene sets related to inflammation and activated immune response. The heatmap in (**C**) collects a series of immunity related genes and indicates upregulation of HLA type II receptors, of their regulator *CIITA* and of immune checkpoint inhibitors *LAG3* and *IDO1*. Please note that inflammatory expression characteristics strongly associate with CD-diagnostics using the Marsh-score: The vertical dashed line divides CD versus non-CD cases (except one Marsh 3A biopsy). (**D**) Profiles of gene sets characterizing functions of the healthy villi (upper crypt), and of basal (lower) crypt taken from [32] indicate antagonistic expression changes from R towards CD-H groups due to progressive villous atrophy.

**Figure A2:**
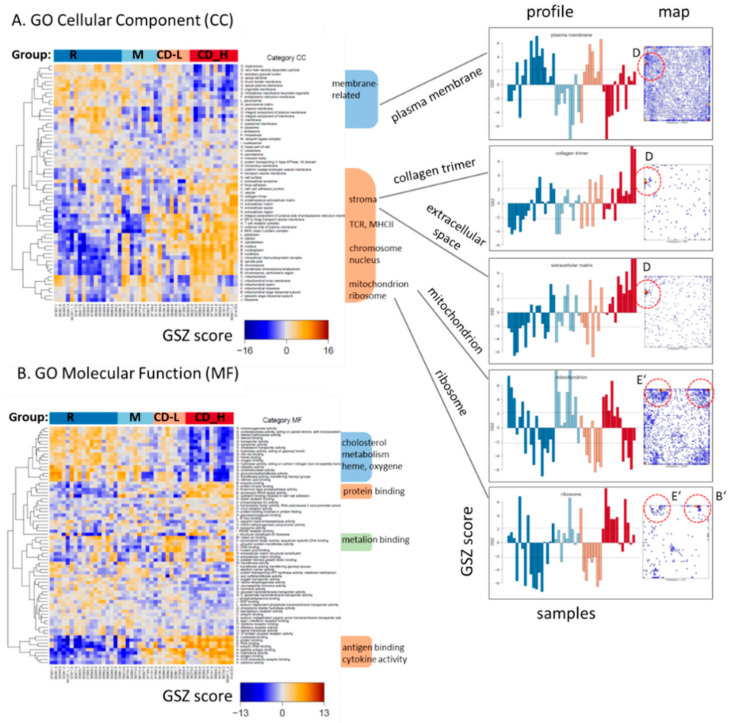
Gene set expression signatures of the gene ontology categories GO (gene ontology) (**A**) CC (cellular complement) and (**B**) GO MF (molecular function) complement functional characterization of CD-types (Figure 2). Expression profiles and gene maps of selected gene sets are shown in the right part. Accumulation of genes in and around spot D associates with high expression in group CD-H (e.g., sets extracellular matrix and collagen trimer). Accumulation in/around spots E’ and B’ increased expression in group M and decays it in CD-L (sets mitochondrion and ribosome) while spot C associates with an antagonistic patterns (set plasma membrane). Gene sets were taken from [29] and implemented in oposSOM [65].

**Figure A3:**
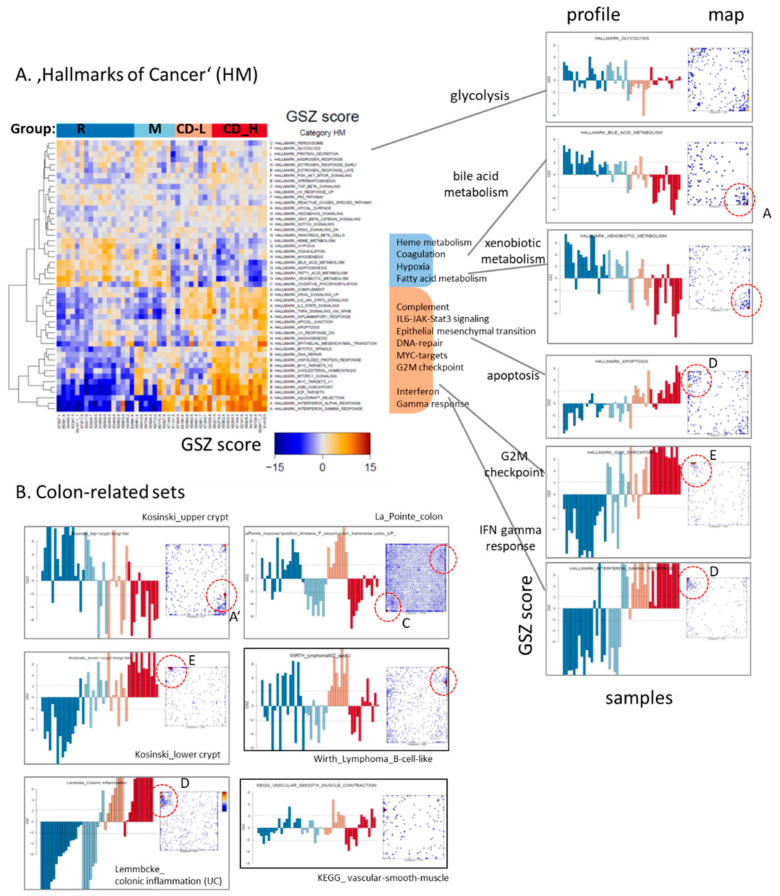
Gene set expression signatures of (**A**), the category hallmarks of cancer (HM, [70]), which reflect general disease-related functionalities activated either in groups R and M (blue background) or CD-L and/or CD-H (red background). Inspection of profiles and spot enrichment of selected gene sets reveals differences between spot D and E: In the former case (set IFN gamma response) one finds activation of CD-cases in groups M, CH-L and CH-H while in the latter case (set G2M checkpoint) only samples from CD-H show activated expression. (**B**) Gene sets taken from colon and lymphoma studies reveal characteristics of colon tissue specimen. Signature genes of the upper and lower crypt [32] accumulate in/near spot A/A’ and E (also reflecting high cell cycle activity), respectively. Colonic inflammation (derived from Ulcerative Colitis versus not-inflamed colon) activates genes in/near spot D and preferentially in CD-L and CD-H samples [27,28]. Interestingly, expression signatures from the healthy colon [35] partly show specific activation in CD-L and deactivation in M-samples. A similar profile is obtained from B-cell lymphoma specimen characterizing a weakly cancerogenic B-cell phenotype presumably due to contaminations of lymph-vessel tissue in the lymph node biopsies [24]. The specific upregulation of the signature KEGG vascular smooth muscle together with the other signatures let us conclude that CD-L biopsies are contaminated by tissue components such as lymph-vessels of lamina propria and eventually mucosa muscularis and submucosal components also present in the colon.

Next we performed functional analysis of the transcriptome data using gene sets related to the functional categories gene ontology (GO) biological process (BP) [29] and to immune response characteristics [30] (Figure 2A,B, see also Figure A2 and Figure A3 for further functional categories). Subgroups R, M and, to a less degree, CD-L show high expression of gene sets reflecting normal metabolic functions of the colon such as flavonoid biosynthetic process, ion transport, oxidative-phosphorylation (OxPhos), cholesterol efflux and cellular glucuronidation. Their expression level markedly drops in CD-H thus indicating the loss of these functions of the healthy intestine. Contrarily, groups CD-H and also CD-L show high expression of inflammatory signatures such as interferon signaling pathways, especially of interferon-ɣ-mediated signaling, antigen processing and presentation, T-cell receptor pathway and positive regulation of T-cell migration, which all indicate activation of the immune system and the infiltration of inflammatory cells into the intestine epithelium in the CD groups in contrast to the R group. Moreover, samples of group CD-H and partly also of CD-L up-regulate processes related to cell division (e.g., the gene sets DNA replication, nucleosome assembly, mitotic nuclear division and cell cycle, Figure 2A). These functions can be interpreted as molecular indicators of crypt hyperplasia and villous atrophy representing characteristic features of coeliac disease [31], which is associated with villous flattening in the small bowel and the loss of digestive functions. In support of this result, we find that the expression level of a gene signature of the bottom ‘lower’ crypt showing highly proliferative characteristics [32] progressively increases from M via CD-L towards CD-H groups, while the expression of a gene signature of the healthy villi with characteristics of the ‘upper crypt’ decays (Figure 2D). The latter set of ‘consisting villi’ includes genes of mucosa and the villi membrane (*AQP10*, *LAMA1*, *MUC12*), as well as genes transcribing for enzymes of normal intestinal functions like *LCT* and *G6PC*. The basal-crypt set includes genes involved in cell proliferation and cell cycle regulation, which are expressed by crypt progenitor cells.

Gene sets of immune function provide further details about the immune response in CD. One finds increasing expression of gene signatures of lymphoid cells such as T-cells, activated T helper cells and cytotoxic T-cells, effector memory CD8+ cells, B-cells and also upregulation of interferon mediated pathways (Figure 2B). The profiles of, e.g., activated CD4+ and of other T-cells further show subtle fine structures. The latter profile anti-correlates with the profile of the ‘healthy villi’ signature which indicates association between the integrity of the colon mucosa and its immune state in agreement with [33]. Expression profiles of a series of immunity-related genes revealed activation of HLA class II antigen presentation in CD paralleled by up-regulation of their regulator (*CIITA*) and of the immune checkpoint inhibitors *CTL4*, *IDO1*, *LAG3* and *PD-L1*. (Figure 2C). Interestingly, the majority of gene signatures related to immune response and of HLA-genes show markedly increased expression levels in biopsies of patients with diagnosed CD and Marsh-scores of 3B/C thus separating CD and non-CD cases also in the mixed group M (see the vertical dashed line in Figure 2A,C and below for further discussion). Gene sets characterizing type 2 epithelial-mesenchymal transition (EMT2) associate with chronically inflamed tissue [34]. We find high activity of EMT2 signature in the CD-H subgroup, which associates with inflammation and possibly leads to fibrosis (Figure A5).

**Figure A4:**
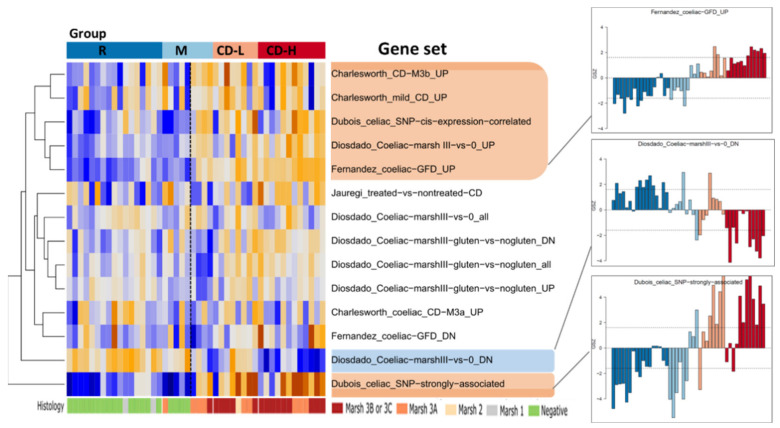
Heatmap of CD-expression signatures taken from previous publications. The signatures were developed for Marsh-score discrimination [13], to characterize villous atrophy [14], epithelial destruction [17], upregulation of immunity via the NFκB pathway [18] and of genes mutated in CD [21]. Overall we observed marked expression differences of cases with diagnosed CD especially in our CD-L and –H groups on one hand and the cases without diagnosed CD on the other hand where most signatures show increasing expression with CD severity (red marked) while one shows the opposite trend (blue background) due to the different selection of genes. Overall, the mapping of literature signatures to our data confirms mutual correspondence.

**Figure A5:**
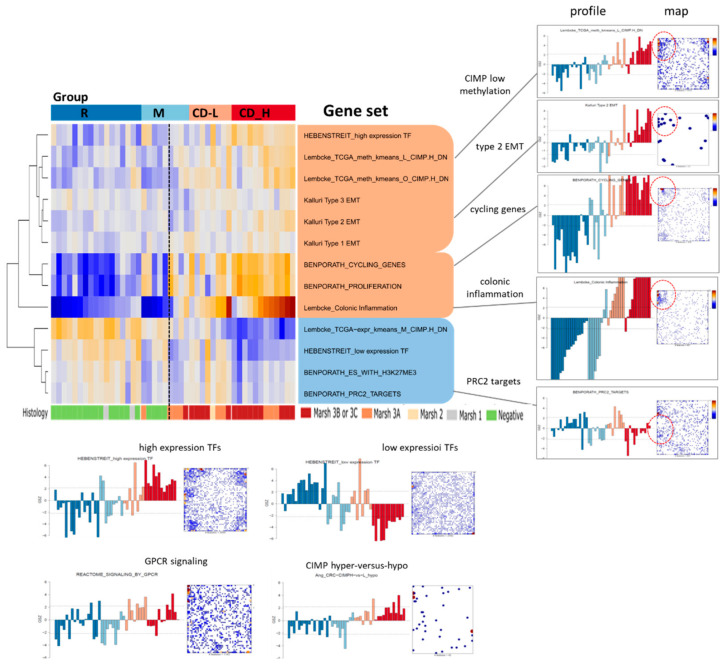
Heatmap of gene expression signatures which associate with transcription factor (TF)-activity and chromatin state [44], type of the epithelial-mesenchymal transition [34], different cellular programs [71] and colonic inflammation [27,28]. Selected signatures were characterized by profiles and gene maps. Gene signature characterizing type 2 epithelial-mesenchymal transition (type 2 EMT) associate with chronically inflamed tissue [34]. Indeed, we find high activity of the type 2 EMT signature in the CD-H subgroup, which associates with inflammation and possibly leads to fibrosis. The high/low expression TF signatures refer to two different modes of genomic regulation which are governed either by TF-binding/debinding in regions of open, actively transcribed chromatin or by epigenetic mechanisms which act via chromatin re-modelling, DNA-methylation and/or histone modifications. Genes related to low expression TFs are activated in the R-group while high expression TFs refer more to CD-H biopsies which suggests a switching of genomic regulation from a more epigenetically driven towards a TF-driven regime (see also [57]). This interpretation is further supported by the expression of PRC2-targets and H3K27me3-repressed genes in the R-group, which reflects epigenetic mechanisms, while high proliferative activity associates with the TF-related regime. Note that expression of cancer-related DNA-methylation CIMP (CpG island methylation phenotype)-signatures suggest loss of DNA-methylation in CD. Affected genes depend to a high degree on the tissue type and thus they presumably apply also to non-neoplastic colonic tissues. Note also that high-TF genes accumulate in regions of spots upregulated in CD-cases, which is not observed for low-TF genes.

**Figure A6:**
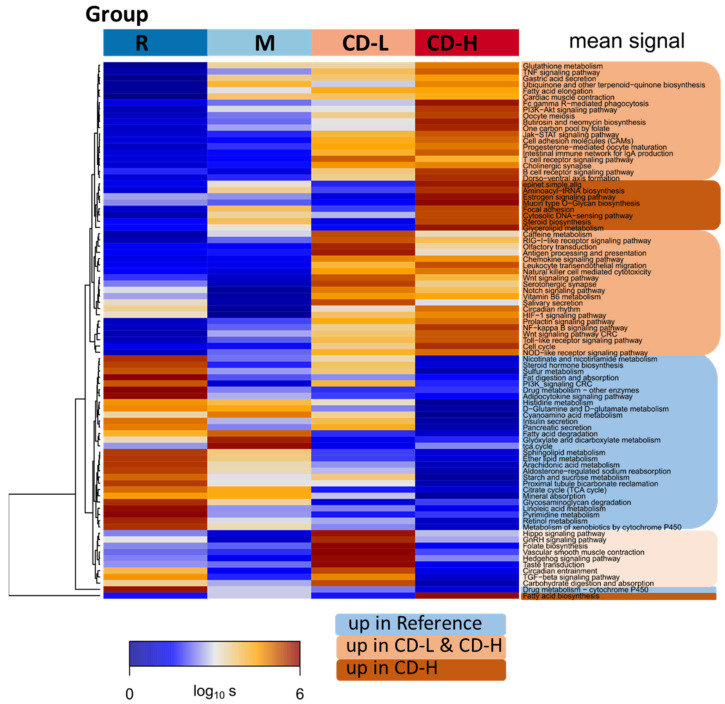
KEGG-pathway activation in the different CD-groups was estimated using pathway-signal-flow (PSF) method [62].

Interestingly, expression signatures from the healthy colon [35], of lymphoma biopsies [24], of the signature KEGG vascular smooth muscle (Figure A3B), G-protein coupled receptor signaling (Figure A5, see also Figure A6 for pathway activation across the groups), of plasma membrane (Figure A2), of signal transduction, apoptosis and microvillus assembly (Figure 2) show specific activation in CD-L and deactivation in M-samples. Overall, the combination of these signatures can be interpreted as a mixture of epithelial, vascular and smooth muscle characteristics possibly originating from contaminations of lamina propria and eventually mucosa muscularis and submucosal components in the biopsies as an alternative option in addition to the indications of low CD severity. We also surveyed expression signatures taken from previous studies on CD which were developed for Marsh-score discrimination [13], to characterize villous atrophy [14], epithelial destruction [17], upregulation of immunity via the NFκB pathway [18] and of genes mutated in CD [21] (Figure A4). Overall, we observed marked expression differences between our CD-L and –H cases on one hand and the R-group on the other hand in agreement with those studies.

**Figure 3:**
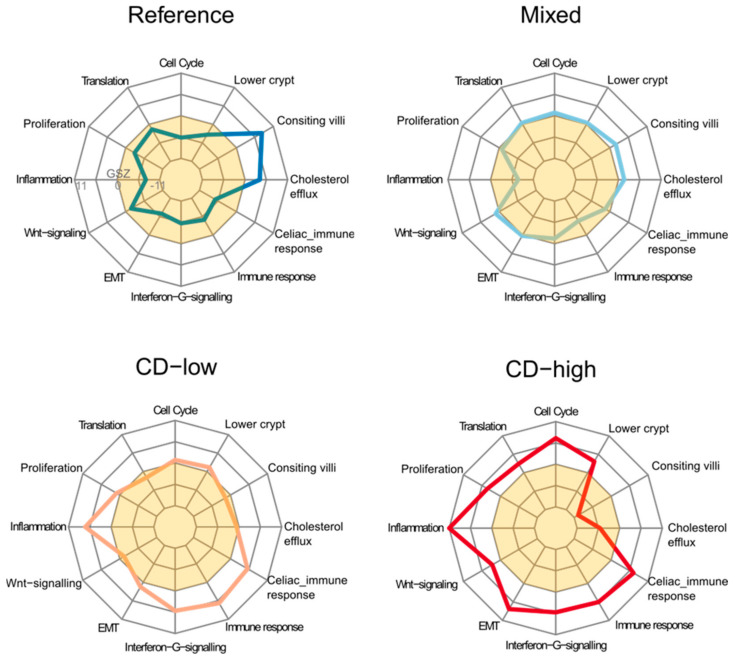
Polar ‘fingerprint’ diagrams of the expression status of CD-types. Each of the axes refer to one gene set. Expression values were scaled in units of the gene set Z (GSZ)-scores estimating mean expression of the gene set in each group divided by its standard variation. White and yellow areas indicate over- (GSZ > 0) and under-expression (GSZ < 0) compared with the mean expression averaged over all samples studied. All CD-diseased groups were characterized by loss of healthy colon functionalities (‘consisting villi’), increased inflammation and proliferative characteristics compared with reference.

The polar diagrams in Figure 3 provide an overview about the major functional characteristics of the four groups of biopsies identified. ‘Consisting villi’ functionality decays in all three CD-related groups but especially in CD-H opposed by gain of different immune-related, inflammatory, type 2 EMT and proliferative function. Taken together duodenal tissue samples from CD patients show concerted changes of expression signatures indicating loss of digestive functions of the duodenum and of villous atrophy paralleled by increased immune response.

### 2.3. Immune Cell Infiltration

**Figure 4:**
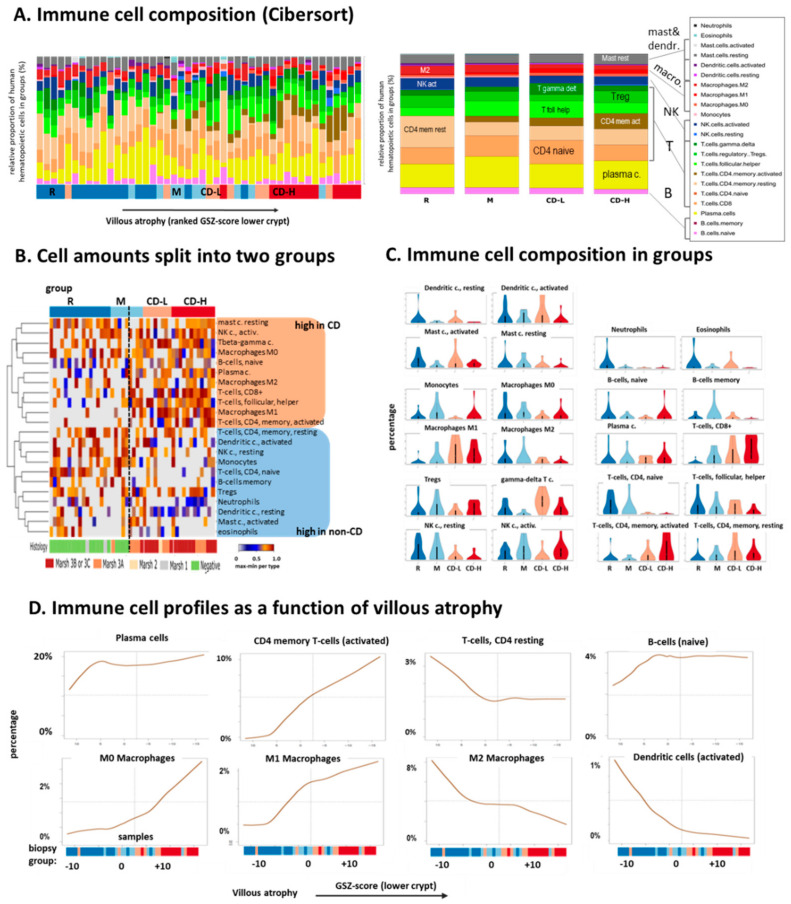
Immune cell infiltration in CD biopsies was estimated using transcriptome deconvolution method [36]. (**A**) The method estimates the relative composition of 22 immune cell-types with single-biopsy (left part, biopsies are ranked with increasing villous atrophy as estimated by means of the GSZ-score of the gene set ‘lower crypt’) and group-wise resolution (right part). (**B**) The heatmap clusters immune cells into two major groups with enhanced amounts in CD or non-CD cases. (**C**) Group-wise immune cell compositions for all 22 immune cell types overall reflect increasing inflammation in CD, e.g., via increased amounts of activated CD4 and CD8 T-cells in CD-L and especially CD-H groups while the amount of resting CD4 and NK-cells decays. (**D**) Villous atrophy profiles of immune cells reveal growing amounts of M0 and M1 macrophages and decaying amounts of M2 macrophages and of dendritic cells. The samples were sorted with increasing GSZ-score of the gene set ‘basal crypt’. The cell type compositions according to Cibersort were then fitted using smooth LOESS functions to obtain cell type compositions as a function of basal crypt transcriptional activity serving as measure of progressing villous atrophy.

Our expression data refer to ‘bulk’ samples representing mixtures of different cell types present in the duodenum biopsies including epithelial cells, fibroblasts and infiltrating immune cells. The composition of immune cell types can be deduced by means of cell-deconvolution methods based on cell-specific transcriptome signatures derived from independent experiments. We here applied “Cibersort” to estimate the relative amount of 22 immune cell-types in each of the biopsies [36] (Figure 4A). Largest fractions refer to plasma cells, naïve and memory resting CD4+ T-cells, T follicular helper and T regulatory cells, however with variable fractions between the different sample groups. Immune cells roughly divide into two groups of either increased or decreased relative amounts in the CD groups compared with the R-, and partly, M-type biopsies (Figure 4B). The former group includes activated T-cells (CD8+ and CD4+) and activated natural killer cells (NK). Stratification of the amounts of immune cells according to the biopsy groups show further details (Figure 4C). The fractions of activated CD4+ and of CD8+ T-cells increase with CD severity while that of resting natural killer cells decays, which, overall, reflects increasing inflammation. CD is characterized by the presence of gluten-specific CD4+ T cells in the lamina propria and by a prominent intraepithelial T-cell infiltration in the epithelial layer that promotes the development of small-intestine inflammation [37]. The proportion of γδT cells was found to slightly increase in CD-L in comparison to M and R. It was previously shown that γδT cells are elevated in the duodenal epithelium of CD patients in comparison to controls using flow cytometry [38,39] providing a potential marker in patients where diagnosis is not straightforward. Further, in CD patients on a gluten-free diet, exposure to gluten induces the appearance of activated, gut-homing CD8^+^ and γδT cells in the peripheral blood [40]. The proportion of those cells is high in CD-L and CD-H. Infiltration of NK cells decay in inflamed duodenal tissue of active CD in agreement previous studies [41].

Next, we ask how immune cell composition changes as a function of villi atrophy. After sorting biopsies with increasing GSZ-expression score of the lower crypt signature (see Figure 4A, left part, and Figure 2D) we fitted the percentages of selected immune cells by smooth continuous functions (Figure 4D). While the relative amount of plasma and B-cells remained relatively constant, we find strong enrichment of CD4 memory cells with progressive atrophy. Interestingly, the fraction of M0 and M1 macrophages gains while the amount of M2 macrophages and of dendritic cells decrease. Macrophages are plastic cells whose phenotype and function are continuously shaped by the surrounding microenvironment that can drive them to acquire a pro-inflammatory M1 or anti-inflammatory M2 phenotype [42]. Our data show that increasing severity of CD has a pro-inflammatory effect on macrophages driving their differentiation toward the inflammatory M1 phenotype in correspondence with previous results showing that the coeliac epithelium modulates macrophages response to gliadin [43]. Overall, these results highlight the importance of the interaction between immune cells and intestinal microenvironment in CD pathogenesis and suggests an orchestrated response of multiple players including epithelium, different types of T-cells, macrophages and of the immune cells which may contribute to the development and progress of the disease.

### 2.4. Transcriptome Portraits Dissect the Expression Landscape into Modules of Co-Regulated Genes Characterizing CD

**Figure 5:**
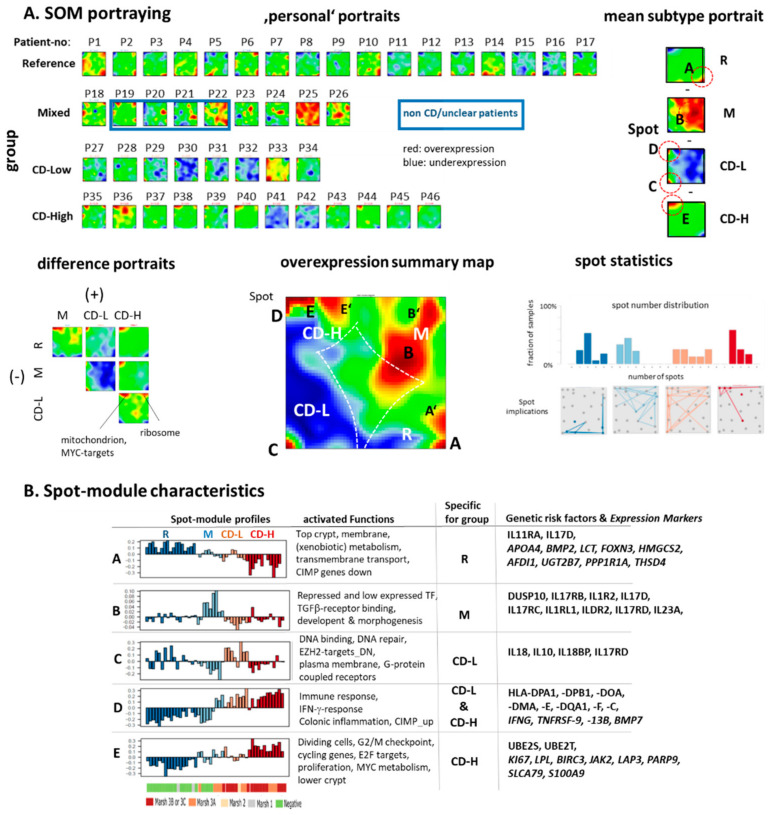
SOM portraying of the expression landscape of CD. (**A**) Individual expression portrays of patients P1–P46, mean group portraits and difference portraits indicate characteristic spot patterns reflecting modules of co-regulated genes. Group specific overexpression spots (red) are indicated by red dashed circles. The portraits are color coded between red (maximum) and blus (minimum) expression. The difference portraits between the groups reveal progressive overexpression of the spots in the left upper corner with increasing CD-severity. Non-CD patients (P19–P22, where diagnosis of P20 is unclear) in the mixed group are indicated by the blue frame. Maroon-to-blue colors in the portraits indicate high-to-low expression levels of the genes located in the respective areas of the images. The overexpression summary map shows five major (labels A–E) spots observed in the sample portraits. Spot statistics describe the spot number distribution of each group and the mutual appearance of spots. CD-H expression portraits show typically only one spot while the broad spot distribution of CD-L reflects a more heterogeneous expression landscape. Additional spots A’ and B’ are found primarily in non-CD specimen of group M (see text). (**B**) Expression profiles of the spots, their functional context, associated risk factor [1] and CD-specific [51] genes characterize the expression modules. Full lists of genes in the spots are provided in the Supplementary Material, Table S1.

Self-organizing map (SOM) machine learning was applied to transform the gene expression data into individual SOM-portraits for each sample. They enable a detailed “personalized” evaluation of the transcriptome landscapes and the identification of modules of co-regulated genes appearing as (red or blue) spot-like areas in the portraits (Figure 5A). One sees that the portraits of the biopsies of the M-group resemble each other independent of their CD-diagnosis. To obtain representative group-wise expression patterns, we also generated mean portraits for each group and differential portraits to highlight differential spot patterns between all combinations of groups (Figure 5A, second row). Overall, five major clusters of co-expressed genes were identified and labeled with capital letters A to E. An overview about the transcriptional landscape is provided by the summary map together with the functional context of the spot-modules (Figure 5B). The CD-H subtype overexpresses spot-modules D and, specifically, E, which accumulate genes involved in immune response and cell cycle activity, respectively. The CD-L subtype upregulates module C, which associates with functions such as DNA binding, DNA repair, apoptosis and also plasma membrane characteristics including G-protein coupled receptor signaling (Figure A2 and Figure A5). Both CD subtypes were characterized by the shared activation of immune response functions (spot D) and an increased proliferation rate, where the CD-L and CD-H subtypes overexpress genes related to DNA-processing and cell cycle activity, respectively. The mixed (M) subtype is characterized by TGFbeta-receptor binding and upregulation of genes associated with repressed chromatin states [44]. They accumulate in spot-module B, which suggests transcriptional regulation by epigenetic mechanisms. Overall, this group shows weaker inflammation and immune response especially of the non-CD cases. The reference group overexpresses spot module A related to healthy colon functions such as xenobiotic metabolism and transmembrane transport processes as for instance cholesterol efflux. Spot-module A is consistently downregulated in all other groups. Moreover, the reference group shows low inflammation and cell cycle activities.

Beyond the major spots A–E we found a few ‘satellite’ spots such as E’ (upregulated signatures mitochondrion, poly-A-RNA binding), B’ (ribosome, translation) and A’ (upper crypt) further detailing cellular mechanisms of M and R groups, respectively. The healthy villi and lower crypt profiles (Figure 2D) closely resemble the profiles of spots A and E, respectively. Gene maps of signature genes indeed accumulate in the respective spot areas (Figure A3B). Moreover, we find *APOA4*, a lipid-processing gene highly expressed in intestinal villi, in spot A and *Ki67* a broadly used cellular proliferation marker expressed in the intestinal crypts [15,16] in spot E. Previous studies have shown that the expression ratio *APOA4*:*Ki67* correlates well with the degree of villous atrophy what makes it a molecular marker for this feature [45,46].

The spot-modules harbor different genes with increased genetic risk for CD [1], which reflects their transcriptional co-regulation together with other genes (see table in Figure 5B). The ‘inflammatory’ spot D contains a series of type I and II HLA genes which upregulate in concert with CD severity, among them *HLA-DQA1* involved in gluten recognition and CD4+ T-cell presentation [47,48]. Spot D also contains the genes *IFNG*, *TNFRSF9* and *TNFSF13B* previously suggested as mRNA blood markers of CD [16]. Additionally, a series of interleukin-coding genes locate in the spot clusters reflecting their strong regulation between the different samples groups. *DUSP10*, a CD-associated candidate gene from genome wide linkage studies involved in proliferation, oxidative stress and innate immunity co-regulates with spot B [49]. The proliferative spot E also contains genes coding ubiquitin-conjugating enzymes [50]. Hence, SOM portrayal enables comparison of samples and subgroups by simple visual inspection of their expression patterns, which, in turn, associate with different molecular functions related to CD pathophysiology.

Spot statistics counts frequency of spot detection in the individual portraits (Figure 5B, part below). The transitory groups M and CD-L show broader distributions compared with the R and CD-H groups, which reflects more heterogeneous expression patterns. Spot implication analysis highlight connections between spots, which were frequently observed together in the same biopsy portraits. This way, different areas of the expression landscape can be assigned to the different sample groups according to spot activation as indicated in the summary map (Figure 5B). Taken together, the gene expression patterns of the about 47,000 transcripts per sample were visualized in terms of “personalized” transcriptomic portraits which, provide a transcriptome landscape of CD-biopsies from the duodenum. It reveals different modules of co-expressed genes up- or down-regulated in the different sample groups together with their functional context.

### 2.5. Expression Signatures and Markers of Coeliac Disease

**Figure 6:**
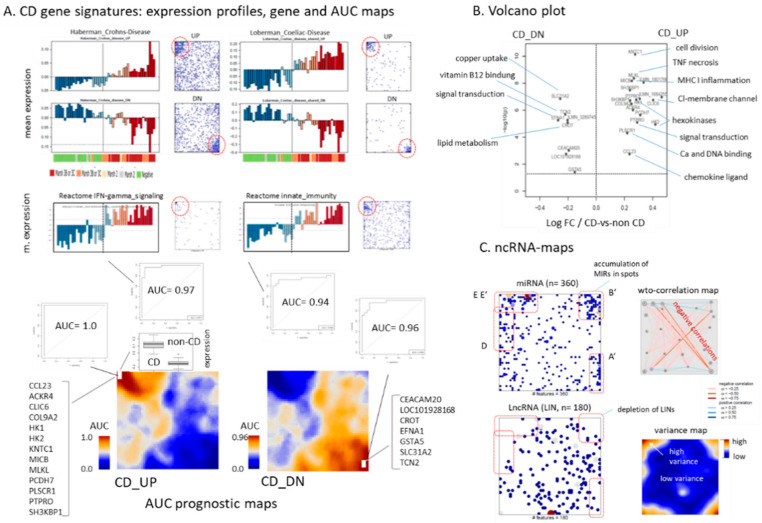
Prognostic expression signatures: (**A**) Expression profiles (mean log-expression of all genes as a function of biopsies) and gene maps (genes of the set were shown as dots in the SOM) of prognostic signatures for Crohns disease [52], Coeliac disease [51] and of Reactome sets ‘Interferon Gamma signaling’ and ‘Innate immunity’. Genes of the UP (upregulated in CD) and DN (downregulated in CD) sets accumulate in opposite corners of the map (red dashed circles), respectively. ROC-curves of the reactome sets provide AUC values for classifying CD-versus-non CD cases of 0.97 and 0.94, respectively. The AUC prognostic maps in the part below are SOM landscapes where each pixel (metagene) is colored according to the AUC-value of the genes included for classifying CD-versus non-CD cases. CD_UP genes in the left upper corner provide perfect classification (AUC = 1) while CD_DN gives AUC = 0.96, which refers to misclassification of 4 biopsies. The boxplot compares expression of the CD_UP genes for CD and non-CD cases. (**B**) The Volcano plot identifies top-differentially expressed genes and their functional context. (**C**) miRNA and of long noncoding RNA are mapped into SOM. While miRNA accumulate in the spot areas of highly variant expression lncRNA deplete (red dashed frames). The correlation map indicates negative (red lines) and positive correlations between genes located in different areas of the map. The expression of highly variant genes located in opposite corners of the map anti-correlate as a rule of thumb while genes located along the upper and lower edge of the map mainly correlate.

Two, out of the four sample groups refer exclusively to CD-patients (CD-L and CD-H) and one divides roughly fifty-fifty into CD and non-CD cases (group M, Table 1). Next, we ask about potential expression markers distinguishing between CD- and non-CD-cases independent of their group membership. First we discovered previous prognostic signatures of CD [51], Crohn’s disease [52] and of Ulcerative Colitis [27] in terms of their expression profiles and gene maps (Figure 6A). The genes of the UP (upregulated in CD) and DN (downregulated in CD) sets accumulate in or near spots D/E in the left upper corner and in or near spot A in the right lower corner of the map, respectively (red circles in Figure 6A). The set-profiles resemble the respective spot profiles (compare with Figure 5B) and also that of the functional sets ‘interferon gamma response’ and ‘innate immunity’ (Figure 6A). Receiver operating characteristics (ROC) illustrate their diagnostic capability. For the functional sets they provide area-under-the-curve (AUC)-values of 0.97 and 0.94, respectively, meaning that the CD cases can be well distinguished from non-CD cases based on pre-defined functional gene signatures. Production of pro-inflammatory cytokines, such as interferon gamma (IFNγ) is known as a hallmark of CD [43] transcripts of which appear in marked amounts not only in CD-H but also in CD-L and in the CD cases of the M-group. Additionally, some of the HLA genes are located in spot D (Figure 5B) which separate most CD from non-CD cases (Figure 1C).

For a closer view onto the expression landscape, we generated so-called AUC-maps by calculating ROC-curves for each pixel/metagene of the SOM and colored them according to the obtained AUC value (Figure 6A, part below). Two separate maps were obtained by processing either CD_UP (genes upregulated in CD) or CD_DN (genes downregulated in CD) profiles. We found maximum AUC values for CD_UP and CD_DN profiles in areas in the left upper and right lower corner of the map, respectively, as expected. The AUCs are slightly asymmetrical enabling ‘perfect’ discrimination (AUC = 1) for CD_UP and slightly worse classification (AUC = 0.96) for CD_DN, meaning that activated genes slightly better classify CD than deactivated ones. Note also, that also that no of the CD_UP genes alone classifies perfectly (AUC = 0.95–0.98) as their mean metagene value does (AUC = 1) owing to noise compensation.

Genes from metagenes with maximum AUC values were further processed using differential expression analysis (*t*-test) and Volcano plot visualization (Figure 6B). Top upregulated genes refer to functions cell division (*KNTC1*), inflammation (*MICB*, *CCL23*), signal transduction and membrane channels (*CLIC6*, *PLSCR1*, *PTPR*) and hexokinases (*HK1* and *HK2*) while top downregulated assign to copper uptake (*SLC31A2*), vitamin B12 (*TCN2*) and lipid metabolism (*CROT*) and signal transduction (*EFNA1*) in agreement with the gene set analysis presented above. In summary, biopsies from patients with diagnosed CD can be separated from non-CD patients with high predictive power based on expression data of groups of genes and of single genes as well. IFN-gamma signaling is one characteristics of CD compared with disease controls.

### 2.6. Noncoding RNAs and Epigenetics

Epigenetics comprises three main factors, DNA methylation, chromatin organization governed by histones and a large battery of enzymes and non-coding RNAs (ncRNAs), which all together have important regulatory functions in gene expression. Generally, ncRNAs regulate gene expression at the transcriptional and post-transcriptional levels. The transcriptomes studied comprise protein coding mRNA and also non-coding RNA such as miRNA (miRs) and long noncoding intergenic RNA (LINs). The 360 MIRs and 180 LINs available on the microarrays used in this study were mapped into the SOM in order to evaluate their co-expression patterns (Figure 6C). The miRs accumulate in distinct spot areas (spots D, E, E’, B’ and A’, see red frames in Figure 6C) which represent clusters of co-expressed mRNA in first instance. miRs are short (17–25 nucleotides), single stranded, highly conserved RNAs that regulate gene expression mostly by inhibiting mRNA translation or by degrading mRNA via specific, sequence-mediated binding [53]. Such repressive interactions between miRs and their mRNA targets imply anti-correlated expression profiles, which appear in the SOM predominantly between transcripts located in opposite corners of the map (see the correlation map in Figure 6C) [54]. Anti-correlative relations suggest that, e.g., miRs located in spots D, E and E’ potentially repress certain mRNA located in or near spots A and A’ and vice versa. Under functional aspects this means that miRs in the former spots are potential repressors of functions associated with the latter spots and vice versa. Previous studies suggested miRs as an importing regulatory layer in CD especially affecting functions such as proliferation, WnT-signaling and cell differentiation associating with spots E, E’ and A, A’, respectively ([55] and references cited therein). miRs found in these spot areas were listed in Table A1 (full lists of ncRNAs in the spots are given in the Supplementary Material, Table S2.).

Surprisingly, the LINs deplete in the spot areas of the SOM. This contrasting distribution compared with that of the miRs (and mRNA) reflects a smaller variance (in units of microarray signal intensities), which prevents localization of the LINs in the highly variant spot areas (see the variance map in Figure 6C). The reason for this difference is not clear. LncRNAs are transcribed from all over the genome acting often as co-factors forming complexes with chromatin modifying proteins and recruiting them to specific sites in the genome, thereby modifying chromatin states and influencing gene expression [56]. Hence, one possible interpretation of the asymmetry between miRs and LINs could be that LINs predominantly act as mediators for subtle adjustments of the transcription under control of epigenetic mechanisms where small changes of abundance can have large effects on mRNA expression. In contrast, miRs act more directly as co-factors mRNA translation and degradation, which requires higher abundances roughly equal to that of mRNA giving rise to higher variability of miR expression.

**Figure A7:**
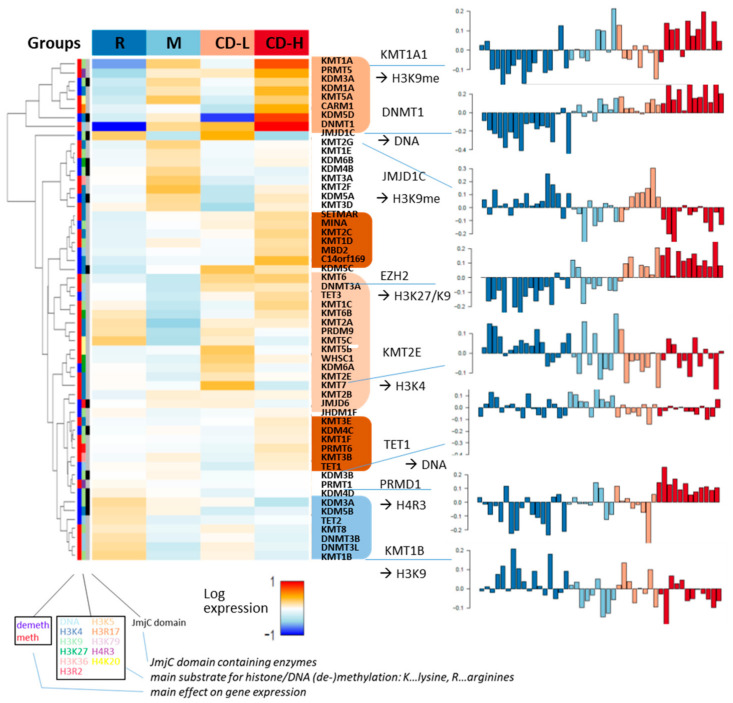
Expression heatmap of chromatin modifying enzymes in the four biopsy groups: The gene expression data of methyltransferases (MTs) and demethylases (DMs) of DNA cytosines, histone lysine (H3K) and arginine (H3R) side chains (see the vertical color bar at the left edge of the heatmap) split into several clusters due to concerted activation and de-activation patterns in the different groups. Clusters upregulated in CD-H and/or CD-L are indicated in brown/dark brown and clusters upregulated in the R-group in blue. Selected expression profiles were shown with single-sample resolution in the right part.

Gene sets referring to these two different modes of gene regulation, i.e., which more directly or more indirectly affect transcription (high- and low-expression TFs, [44,57]) show a similar mutual accumulation-depletion dualism in the areas of spot upregulated in CD (Figure A5) as the miRs and LINs. Moreover, genes related to low expression TFs are activated in the R-group while high expression TFs refer more to CD-H biopsies which suggests switching of genomic regulation from a more epigenetically driven towards a TF-driven regime (see also [57]). This interpretation is further supported by the expression of PRC2-targets and H3K27me3-repressed genes in the R-group, which reflects epigenetic mechanisms via histone modifications and DNA-methylation, while high proliferative activity associates with the TF-related regime (Figure A5). We also compared the expression levels of more than 50 epigenetic modifiers (genes coding enzymes which write, read or erase histone modifications and/or DNA methylation marks, see [58] for an overview) between the biopsy groups and found considerable deregulation of the epigenetic machinery in CD compared with the disease controls (Figure A7) presumably affecting chromatin organization and predicting widespread alterations of DNA methylation in CD. In support of this expectation we found that CIMP (CpG island methylator phenotype) genes, which are prone to promoter hypermethylation in colon cancer subtypes [27,59] accumulate in and around spot D (Figure A5), which suggests their demethylation in CD due to anticorrelation between promoter methylation and expression of CIMP genes [27], in partial agreement with DNA-methylation studies on CD biopsies [60]. In summary, miRs form a strong regulatory layer associating with highly variant mRNA expression modes in contrast to LINs showing smaller expression changes between the biopsies, possibly because of their role in more subtle epigenetic modes of transcriptional regulation. Massive expression changes of the machinery of chromatin modifiers implies that CD is paralleled by chromatin remodeling and DNA-methylation changes.

### 2.7. Browsing the Transcriptome of Coeliac Biopsies

**Figure 7:**
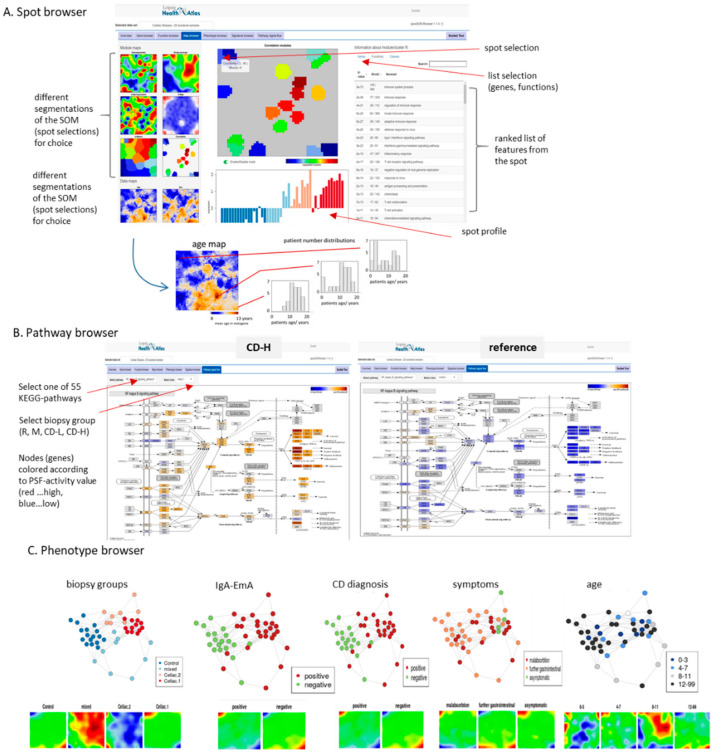
The interactive data-browser enables detailed study of different aspects of the CD-transcriptome dataset [61] (https://www.izbi.uni-leipzig.de/opossom-browser/?dataset=10 (accessed on 3 March 2021)). (**A**) The map browser (shown as screenshot) provides feature (genes, gene sets) lists and profiles of selected spot-areas of the SOM. The age phenotype map colors metagene expression as a function of the underlying patients’ age. Blue indicates younger and maroon elderly patients. (**B**) The pathway browser provides activity patterns for each of the biopsy groups. The screenshot shows the NFκB pathway for CD-H and R groups. (**C**) The phenotype browser visualized the sample similarity net and mean SOM-portraits according to different stratification criteria.

**Figure A8:**
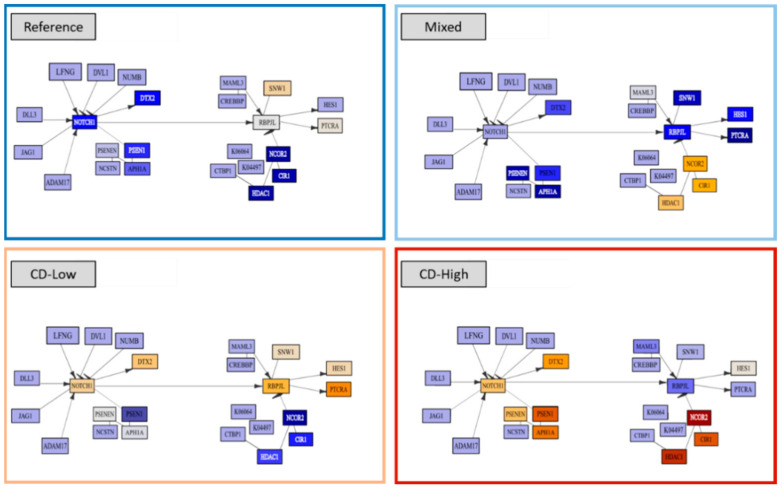
Pathway activation patterns of the NOTCH pathway in the different CD-groups. In healthy tissue, genes for transmembrane receptor protein Notch 1, 2 and 3 are highly expressed at the basal crypts of morphologically normal colon mucosae [32]. NOTCH1 interacts with the Wnt-pathway and activates cell proliferation. We find elevated mRNA expression of NOTCH1 in subgroups CD-H and CD-L (FC 1.38, *p* < 0.01) compared to Reference samples. Interestingly, the activation patterns of the NOTCH-pathway varies in a group-specific fashion. The genes PSENEN, PSEN1 and AP1A activate specifically in CD-H (and to a less degree in M) while DTX2, RBPJL and PRCRA were found activated in CD-L.

For more detailed interactive discovery of the dataset presented in this publication we offer the oposSOM-browser platform (https://apps.health-atlas.de/opossom-browser/?dataset=10 (accessed on 3 March 2021)) [61]. It consists of an overview window and six ‘sub-’browser functions. The “gene-” and “function-” browsers enable to select single genes and functional gene sets to display their profiles and gene localization in the SOM landscape. The “signature” browser shows the same features for single genes or lists of genes, and, in addition, their classification quality for selected sample strata in terms of ROC-curve and AUC-value. As an example one finds for the gene *IFNG* perfect separation between CD and non-CD cases (AUC = 1), nearly as good separation for ImA-EmA positive and negative cases (AUC = 0.95) but no differentiation between male and female patients (AUC = 0.41) as expected. The “spot-” browser allows to discover different segmentations of the SOM into spot modules of co-regulated genes to list the genes included, their functional context and the sample groups where they are activated (Figure 7A). The spot browser also shows age- and sex-maps displaying associations between age or sex with gene expression levels in the map. For example, the age-map reveals higher prevalence of spot D and E (up in CD-H) in younger patients compared with the R-group (Figure 7A). The “pathway-” browser enables us to select one of 55 KEGG-pathways and to visualize its gene-activation in each of the biopsy groups as estimated using the pathway signal flow algorithm [62]. For example, the NFκB signaling pathway reveals marked activation in CD-H compared with the reference group (Figure 7B, see also Figure A8 for the NOTCH pathway). The “phenotype-” browser allows selection of different biopsy characteristics such as CD-diagnosis, sample group or IgA-EmA status to inspect the respective sample similar network and group-averaged SOM-portraits (Figure 7C). One sees, for example, that CD-diagnosis and IgA-EmA status give rise to nearly identical group portraits (see also Figure 1A), while with increasing age CD-related expression in younger patients increases. Hence, the browser-functionality enables interested scientists to discover details of the data with higher resolution and/or under special perspectives not explicitly addressed in this publication.

## 3. Discussion

**Figure 8:**
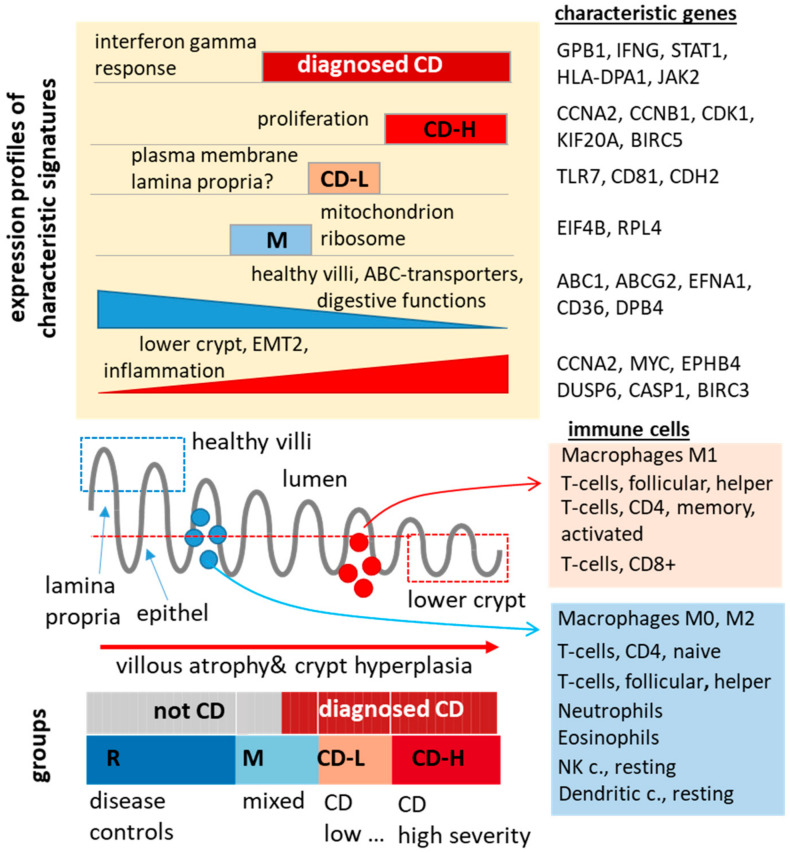
Transcriptomic heterogeneity of coeliac biopsies from the duodenum. See text.

We find considerable heterogeneity of the transcriptomes obtained from biopsies of the duodenum, which were collected from 24 young (age 3–18 years) CD patients, from 21 non-CD patients with different gastrointestinal complaints serving as disease controls, and one 10 years old male patient with unclear diagnosis. Their whole transcriptome expression landscapes were stratified into four groups using unsupervised clustering, namely a reference group (no CD cases), a mixed group (about 50% CD) and two groups comprising only CD cases assigned as CD-L (low) and CD-H (high). An important factor of variation between the groups associates with the relative amounts of lower crypt and consistent villous (upper crypt) epithelial tissue in the biopsies, each having its specific transcriptomic characteristics dominated by signatures reflecting either high levels of cell proliferation or digestive (e.g., lipid and xenobiotic metabolism) functions (see Figure 8 for illustration). In agreement with previous studies [11,12,13,14,15,16], we find the progressive increase in lower crypt and the decrease in upper crypt characteristics in direction from R, via M towards CD-L and CD-H groups as an indication of villous atrophy and crypt hyperplasia representing a measure of CD severity. Moreover, the pro-inflammatory character of CD is demonstrated by increasing gene expression of a large battery of inflammation signatures along this severity axis in correspondence with standard serum markers, which were measured independently.

Some of these signatures alter rather smoothly in this way, forming a continuum of activated states (e.g., xenobiotic metabolism), while others change more discontinuously with clear-cut borderlines between the biopsy groups. For example, proliferative characteristics (e.g., cell cycle activity) markedly increase in CD-H in comparison with CD-L possibly as an indication of virtually complete villous atrophy, giving rise to the drop of upper crypt and, in parallel, the steep increase in the lower crypt proliferative expression signature. On the other hand, both ‘intermediate’ groups show specific signatures in addition to the intermediate levels of inflammation and villous atrophy observed in these groups. Particularly, group M is characterized by the upregulation of mitochondrial and ribosomal transcriptional programs possibly in response to unspecific gastrointestinal complaints. Group CD-L, on the other hand, specifically activates gene signatures of the healthy duodenum [35] and of the lymphatic system [24] possibly reflecting contaminations of lamina propria, vascular and other tissues in the biopsies (Figure A3B).

We found that about 50% of all miRNA expression values available in the transcriptome are highly variant between the groups thus providing an interesting reservoir of potential markers for the different biopsy groups. For the expression levels of long non-coding intergenic RNA we found a completely different, less-variant picture, possibly because LINs predominantly fulfill functions in epigenetic regulation of transcription governed by low-expression transcription factors, in contrast to miRNA which regulates post-transcriptional mRNA levels via direct binding. Further studies are required to better understand this asymmetry of transcriptional variation and also to specify mRNA targets of the miRNA identified here. Note that the microarrays used focus on mRNA while only a selection of non-coding RNA is available for analysis. We found indications of epigenetic mechanisms accompanying pathogenesis of CD such as transcriptional de-regulation of a large battery of epigenetic modifiers and expression changes of genes prone to methylation in the diseased colon.

Immune cell compositions in the biopsies were deduced by cell type deconvolution of transcriptome data using “Cibersort” [36]. Currently, this is the first report defining leukocytes by mRNA expression in biopsies of CD patients without previous cell sorting. We find increasing amounts of pro-inflammatory T-cells in CD-L and CD-H biopsies paralleled by macrophage polarization from M0 and M2 into the inflammatory M1 phenotype. Some immune cells, such as γδ T-cells, activated mast and dendritic cells (Figure 4B), show slightly elevated levels in CD-L, which supports the hypothesis that the tissue composition of this group differs from CD-H. Recent single cell transcriptomic studies reported dramatic changes of the immune cell landscape in the CD lesions, where especially T-cells are transcriptionally different from their control counterparts [63]. In consequence, reference signatures used for immune cell deconvolution only approximately reflect the true cell type composition and must be confirmed experimentally, e.g., by single cell transcriptomics, or improved deconvolution methods.

Thus, CD-biopsies reveal considerable heterogeneity due to different levels of villous atrophy and crypt dysplasia, inflammation and immune response including immune cell infiltration and presumably varying amounts of epithelial and lamina propria tissue. Despite this heterogeneity we find signatures (e.g., interferon gamma signaling) and single mRNA markers (e.g., *IFNG* gene) which reliably distinguish CD from non-CD cases independent of their biopsy-group membership. Larger cohorts and long-term longitudinal clinical data about treatment, diet, lifestyle, and well-being are necessary to increase the robustness of these markers for CD-diagnosis. Otherwise, application of identified CD-specific transcriptional signatures allows clear assignment even of the patient with unclear diagnosis to the group of CD patients. In our study, clinical diagnosis of CD for the unclear patient was not made during the ABCD study period despite noticeable antibody constellation, an HLA-DQ8 haplotype, and severe symptoms. Of 929 patients recruited in the ABCD study [64], 29 (2.5%) obtained no final diagnosis. This underlines the requirement of further improved diagnostic tools. Combining single highly specific mRNA markers for villous atrophy and crypt dysplasia, as well as inflammation or infiltrating immune cells might be a possible approach to close the diagnostic gap.

From a methodical perspective, we, for the first time, applied SOM machine learning to this disease. This method delivers “personalized” transcriptome portraits for each of the biopsy specimens enabling direct comparison and feature extraction by visual perception. Our interactive SOM browser complements analyses presented in this publication by a series of functionalities, which can be selected by interested scientists, e.g., to display expression profiles of single genes, to evaluate their discriminative power for classifying different sample strata or to inspect gene activation patterns of a series of pathways.

A limitation of this study is that biopsies were collected in two children’s hospitals by two different gastroenterologists, respectively. We found that the proportions of patients recruited in one children’s hospital differed strikingly in Group CD-L and CD-H (45% vs. 25%). Divergent procedures concerning the collection of biopsies during upper gastrointestinal endoscopy may result in different transcriptional landscapes of the duodenal tissue biopsies. In addition, biopsy quality (in terms of villus structures, exclusion of samples with lymphoid follicles or Brunner’s glands) was not monitored in all cases prior to freezing. Patchiness of inflammation has been reported in biopsy samples of coeliac disease. Presumably, transcriptional analysis reflects this to a certain degree. Since the routine biopsies used for the Marsh classification and those used for transcriptional analysis are different by nature of the analysis, variation between both types cannot be excluded. On the other hand, the independent type of sampling and the mostly consistent assignment of biopsies mutually supports diagnosis.

## 4. Materials and Methods

### 4.1. Study Design and Patients

Patients were recruited as part of the ABCD (Antibody diagnostics in pediatric Coeliac Disease) study in two children hospitals in Leipzig (Saxonia, Germany). The trial was registered in the German Clinical Trials Register (DRKS00003854). The study protocol, patient information sheets, and informed-consent forms were approved by each site’s ethics committee. Patients aged between 5 months and 18 years were scheduled for duodenal biopsy with primary aim to confirm or refuse coeliac disease. Patients were only included if they had not already been diagnosed with coeliac disease, if they were not on a gluten-free diet (GFD), and if they had not received immunosuppressive therapy within the last eight weeks. For a detailed description of the study design, see [64]. Overall, the ABCD subproject included 46 children and adolescents of whom 24 patients were finally diagnosed with CD (including 5 patients with type 1 diabetes), 21 patients were considered as control patients with different gastrointestinal complaints and one case was not clear (Table 1).

### 4.2. RNA Extraction and Microarray Measurements

For study purpose, one biopsy (15–20 mg) was taken from the descending duodenum of each patient. Fresh tissue samples were snap frozen and stored in liquid nitrogen until preparation. Frozen biopsies were disrupted and homogenized by TissueLyzer from Quiagen (Hilden, Germany). Total RNA was isolated using AllPrep^®^ DNA/RNA Micro kit (QIAGEN, Hilden, Germany) and stored at −70 °C.

Before microarray analysis RNA integrity and concentration was examined on an Agilent 2100 Bioanalyzer (Agilent Technologies, Palo Alto, CA, USA) using the RNA 6.000 LabChip Kit (Agilent Technologies) according to manufacturer’s instructions. Then, 250 ng RNA per sample was ethanol precipitated with GlycoBlue (Invitrogen) as carrier and dissolved at a concentration of 100–150 ng/µL prior to probe synthesis using the TargetAmp™- Nano Labeling Kit for Illumina Expression BeadChip (Epicentre Biotechnologies, Madison, WI, USA). From each probe, 750 ng of cRNA were hybridized to Human HT-12 v4 Expression BeadChips (Illumina, San Diego, CA, USA) and scanned on the Illumina HiScan instrument according to the manufacturer’s specifications.

### 4.3. Antibody Assays and HLA-Typing

Blood samples were collected around the time of the diagnostic duodenal biopsies and used for HLA-typing. IgA-antibodies against tissue transglutaminase (IgA-TTG), IgG-antibodies against deamidated gliadin peptides (IgG-DGP) and IgA-endomysium antibodies (IgA-EmA) were measured in sera with test kits from EUROIMMUN (Luebeck, Germany; cut-offs ≥20 U/mL, ≥25 U/mL, ≥1:10, respectively). If antibody concentrations were above the measurement range, sera were serially diluted and values corrected by the dilution factor. For HLA-typing, genomic DNA was isolated by QIAamp^®^ DSP DNA Blood Mini Kit (QIAGEN, Hilden, Germany). HLA-DQ2.2, -DQ2.5 and -DQ8 was determined applying the EUROArray HLA-DQ2/DQ8 kit (EUROIMMUN, Luebeck, Germany).

### 4.4. Expression Analysis and SOM Portrayal

Raw probe data were quantile normalized, centralized, and then clustered using self-organizing map (SOM) machine learning utilizing the *oposSOM* package [65]. This method translates the gene expression values of over 47,000 transcripts into 2500 *meta-gene* expression values. They were visualized in terms of two-dimensional 50 × 50 mosaic images of each sample using a maroon-to-blue color code for high-to-low meta-gene expression values, respectively. Size and topology of the SOM-portraits were chosen to allow robust identification of expression-modules, called ‘spots’, representing clusters of co-expressed meta-genes in the samples studied [66]. Mean group SOM portraits were calculated by averaging the meta-gene expression values of all cases of the respective group. Difference portraits between groups were calculated as the differences between the meta-gene values of the respective mean group portraits.

### 4.5. Identification of Transcriptional Subclasses

Sample diversity analysis and class discovery were performed as described previously [67]. It includes so-called sample-SOM, which visualizes similarity relations between the samples instead of similarity relations between gene-profiles. Subclasses of samples were obtained by hierarchical clustering in *Euclidian* distance space between the meta-genes state of different samples in combination with silhouette score verification [68]. The silhouette score is defined as the difference between the intra-class and the best inter-class similarity of each sample using Pearson’s correlation coefficient between their meta-gene expression landscapes as measure [68]. The silhouette score is positive for samples which fit well into the cluster chosen, whereas the score is negative for samples which better fit to other clusters.

### 4.6. Functional Analysis

For functional interpretation of the expression-modules, we applied gene set analysis using the gene set Z-score (GSZ) which estimates the activity of a gene set in each sample [69]. Enrichment of gene sets in the modules was calculated applying Fisher’s exact test. We considered gene sets related to biological processes (BP) of the gene ontology (GO) classification, standard literature sets [29], and also literature sets curated by us. To estimate pathway activity, we used pathway signal flow (PSF) analysis as implemented in oposSOM [62]. PSF estimates the signal flow propagation based on gene expression data and the pathway topology. Immune cell deconvolution was performed by means of Cibersort [36]. It provides the relative amount of 22 immune cell types based on transcriptomic data in each of the biopsies.

### 4.7. OposSOM Browser

The results of transcriptome analyses presented in this publication can be interactively discovered regarding further details using the oposSOM browser [61] available on the Internet under https://apps.health-atlas.de/opossom-browser/?dataset=10 (accessed on 3 March 2021). Functionalities include browsing (i) single gene profiles across the biopsy samples and the expression landscape, (ii) gene set profiles, (iii) different types of expression and phenotype maps, (iv) sample and subtype diversity, (v) marker signatures, and (vi) KEGG-pathway activation in the different groups.

## Figures and Tables

**Table 1:** Clinical and histological characteristics of the patient groups ^1^.

Patient Group	Reference	Mixed	CD-Low	CD-High
N (cases)	17	9	8	12
Diagnosis				
Coeliac disease	0 (0.0%)	5 (55.5%)	8 (100%)	12 (100%)
Disease controls	17 (100%)	3 (33.3%)	0 (0.0%)	0 (0.0%)
Unclear	0 (0.0%)	1 (14.2%)	0 (0.0%)	0 (0.0%)
**Age**				
Median (range)	14 y (1–17)	10 y (3–12)	8 y (3–17)	13 y (4–15)
**Age interval**				
1y–4y	3 (17.6%)	1 (11.1%)	2 (25.0%)	5 (41.7%)
5y–8y	1 (5.9%)	2 (22.2%)	1 (12.5%)	1 (8.3%)
9y–12y	3 (17.6%)	6 (66.7%)	1 (12.5%)	1 (8.3%)
13y–17y	10 (58.5%)	0 (0.0%)	4 (50.0%)	5 (41.7%)
**Sex**				
Female	13 (76.5%)	6 (66.7%)	7 (87.5%)	5 (41.7%)
**Gastrointestinal complaints**				
at least one sign of clear malabsorption ^(1)^ asymptomatic ^(2)^	17 (100%)	8 (88.9%)	6 (75.0%)	9 (75%)
	0 (0.0%)	3 (33.3%)	3 (37.5%)	7 (58.3%)
	0 (0.0%)	0 (0.0%)	1 (12.5%)	3 (25.0%)
**Symptoms since** ^(3)^				
<3 months	0 (0.0%)	0 (0.0%)	0 (0.0%)	1 (8.3%)
3–24 months	11 (64.7%)	4 (4.44%)	4 (50.0%)	5 (41.7%)
>24 months	5 (29.4%)	4 (44.4%)	2 (25.0%)	3 (25%)
**IgA-aTTG**				
positive	1 (5.9%)	6 (66.7%)	8 (100%)	11 (91.7%) ^(4)^
**IgG-EmA**				
Positive	1 (5.9%)	6 (66.7%)	8 (100%)	12 (100%)
**IgG-aDGL**				
positive	1 (5.9%)	4 (44.4%)	6 (75.0%)	11 (91.7%)
**HLA-Type**				
negative	10 (58.8%)	2 (22.2%)	0 (0.0%)	0 (0.0%)
DQ2 ^(5)^	7 (41.2%)	6 (66.7%)	7 (87.5%)	11 (91.7%)
DQ8	0 (0.0%)	1 (11.1%)	1 (12.5%)	1 (8.3%)
DQ2 and DQ8	1 (5.9%)	0 (0.0%)	0 (0.0%)	1 (8.3%)
**Marsh stage**				
Normal	15 (88.2%)	4 (44.4%)	0 (0.0%)	0 (0.0%)
Marsh 1	2 (11.8%)	0 (0.0%)	0 (0.0%)	0 (0.0%)
Marsh 2	0 (0.0%)	0 (0.0%)	1 (12.5%)	0 (0.0%)
Marsh 3A	0 (0.0%)	4 (44.4%)	2 (25.0%)	3 (25.0%)
Marsh 3B/C	0 (0.0%)	1 (11.1%)	5 (62.5%)	9 (75.0%)

^(1)^ malabsorption includes chronic diarrhea, weight loss, and failure to thrive. ^(2)^ all asymptomatics were CD patients with type I diabetes. ^(3)^ information about symptoms duration was missing for one patient in the reference group. ^(4)^ one patient with partial IgA deficiency. ^(5)^ includes HLA-DQ2.5 and HLA-DQ2.2. CD-L = low-severity coeliac disease, CD-H = high-severity coeliac disease, IgA-aTTG = IgA antibodies against tissue transglutaminase, IgA-EmA = IgA anti-Endomysium, IgG-aDGL = IgG antibodies against deamidated gliadin peptides, M = Mixed group R = Reference group.

**Figure A1:**
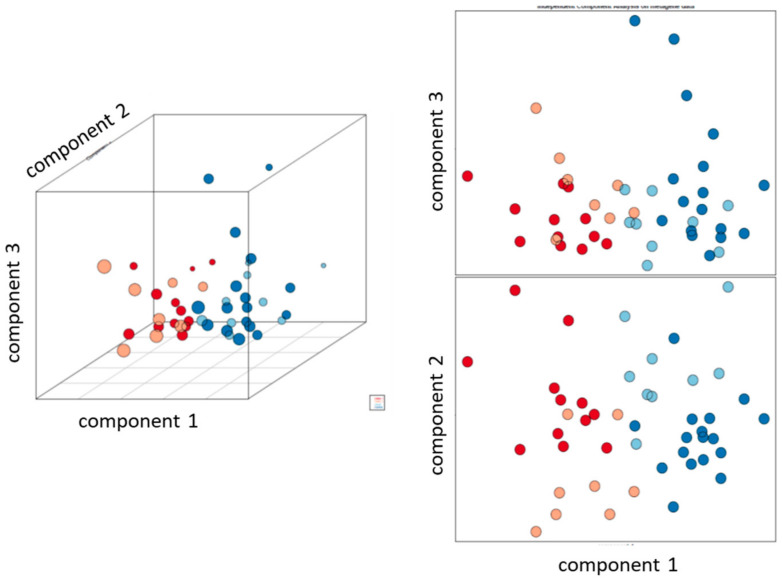
Independent component analysis (ICA) plot of biopsy groups. Component 1 distinguishes virtually the CD-H and R groups while component distinguishes mainly CD-L and M groups. ICA is implemented in oposSOM. Sample SOM plot combines component 1 to component 3 features.

## Data Availability

Expression data are available in the gene expression omnibus (GEO) database under accession number GSE164883.

## Supplementary Materials

The following are available online at https://www.mdpi.com/1422-0067/22/5/2551/s1, Table S1: List of genes included in the spots of co-expressed genes; Table S2: Lists of ncRNA included in the spots.

## Appendix A

Additional expression characteristics.

**Table A1:** miRNA (miRNA) and long intergenic RNA (LIN) located in different spots of the SOM.^(a)^.

Spot	miRNA	LIN	
**A**	miR-34AHG, -555, -4260, -7114, -6778, -4469, -22, -6084, -37146125, -1248	LINC00324 LINC00479	down in CD
**A’**	miR-614, -6887, -6778, -6887, -6872, -6791, -6778, -1199, -7846, -4651, -6729, -4640, -6787, -6836, -4329, -6734, -6721, -4687, -6833	LINC-PINT LINC01547 LINC00319 LINC00909 LINC00852	down in CD
D	miR-4435-2HG, -3606, -198, -5195, -1287, -4649, -5047, -1182, -568, -4720, -7704		Up in CD
E	miR-6732, -7108, -3917, -147B, -25, -664B, -4722, -147B, -636, -7107		Up in CD

^(a)^ miRNAs in spots A and A’ are assumed to repress mRNA and associated functions in spots D and E while miRNA in spots D and E are assumed target mRNA in spots A and A’. In other words, miRNA in spots D and E potentially facilitates development of CD while miRNA in A and A’ acts in the opposite direction.
